# Implementing PSO-ELM Model to Approximate Trolox Equivalent Antioxidant Capacity as One of the Most Important Biological Properties of Food

**DOI:** 10.1155/2021/3805748

**Published:** 2021-08-02

**Authors:** Marischa Elveny, Ravil Akhmadeev, Mina Dinari, Walid Kamal Abdelbasset, Dmitry O. Bokov, Mohammad Mahdi Molla Jafari

**Affiliations:** ^1^DS & CI Research Group, Universitas Sumatera Utara, Medan, Indonesia; ^2^Department of Accounting and Taxation, Plekhanov Russian University of Economics (РRUE), Stremyanny Lane 36, 117997 Moscow, Russia; ^3^Department of Law, Faculty of Economics and Social Sciences, Shahid Chamran University of Ahwaz, Ahvaz, Iran; ^4^Department of Health and Rehabilitation Sciences, College of Applied Medical Sciences, Prince Sattam Bin Abdulaziz University, Al-Kharj, Saudi Arabia; ^5^Department of Physical Therapy, Kasr Al-Aini Hospital, Cairo University, Giza, Egypt; ^6^Institute of Pharmacy, Sechenov First Moscow State Medical University, 8 Trubetskaya St., Bldg. 2, Moscow 119991, Russia; ^7^Laboratory of Food Chemistry, Federal Research Center of Nutrition, Biotechnology and Food Safety, 2/14 Ustyinsky Pr., Moscow 109240, Russia; ^8^Department of Petroleum Engineering, Ahwaz Faculty of Petroleum Engineering, Petroleum University of Technology (PUT), Ahvaz, Iran

## Abstract

In this paper, the Trolox equivalent antioxidant capacity (TEAC) is estimated through a robust machine-learning algorithm known as the Particle Swarm Optimization-based Extreme Learning Machine (PSO-ELM) model. For this purpose, a large dataset from previously published reports was gathered. Various analyses were performed to evaluate the proposed model. The results of the statistical analysis showed that this model can predict the actual values with high accuracy, so that the calculated *R*^2^ and RMSE values were equal to 0.973 and 3.56, respectively. Sensitivity analysis was also performed on the effective input parameters. The leverage technique was also performed to check the accuracy of real data, and the results showed that the majority of data are reliable. This simple yet accurate model can be very powerful in predicting the Trolox equivalent antioxidant capacity values and can be a good alternative to laboratory data.

## 1. Introduction

Many antioxidant compounds may be found in vegetable materials [1, 2]. To find possible sources of natural antioxidants for use in edible products, many plants have been researched and several substances have been identified [3, 4].

Biologists and physicians are also interested in antioxidants because of their use for guarding the human organs against the harm of reactive oxygen species (ROS) [5, 6]. Because of the alleged tight relationship between oxidative stress and illness, antioxidants are thought to be preventive agents against similar illnesses [7, 8].

There is a negative relationship between consuming fruits and vegetables, as the greatest sources of antioxidants, and the cancer risk, so that the risk is reduced by 30-50% [9, 10]. The antioxidant potential of a wide range of substances is investigated by many researchers; the substances include different vegetables and fruits, drinks, green teas and black ones, plant parts (their leaves, the roots, and their bark), kernel, shell, and also industrial wastes and secondary products apart from the ones derived from plants [11]. The “antioxidant capacity” refers to the certain amount of free radicals being removed by a laboratory solution, without considering the properties of any antioxidant of the mix [12, 13].

The antioxidant capacity of the Trolox equivalent of nutritional excerpts is then measured by taking into account the aggregate activity of all antioxidants of the excerpts, together with their chain-breaking, cleansing, and chelating impacts, hence offering an inclusive factor instead of the measurable antioxidants' simple sum [14]. So we can identify the antioxidants' familiar and unfamiliar capacities, and their synergistic relationship, providing a method for identifying a diverse variety of nutrition for antioxidant characteristics [15, 16]. There are many kinds of research for measuring the antioxidant capacity of Trolox equivalent of nutritional products [17–21].

The basis of each technique is producing various free radicals via a range of methods, followed by measuring a variety of endpoints at a defining moment or above the limit [22]. A spectrophotometric test of the antioxidant capacity of the Trolox equivalent is a frequent technique, which has relied on the antioxidants' relative capacities that exist in nutritional products to remove the radical cation of ABTS^+^ (2,2′-azinobis-(3-ethyl-benzothiazoline-6-sulfonic acid)), comparing to the capacity of the antioxidant of 6-hydroxy-2,5,7,8-tetramethylchroman-2-carboxylic acid (Trolox) standard amounts [17, 23]. We previously evaluated the substances of physiologically active chemicals and the cruciferous seeds' Trolox equivalent antioxidant capacity during sprouting, and the findings on ascorbic acid levels were previously reported [24].

The present paper is aimed at developing a PSO-ELM model for forecasting the antioxidant capacity of the Trolox equivalent of various sprouting cruciferous seeds dependent upon the entire phenolic composites, inositol hexaphosphate, glucosinolates, soluble proteins, ascorbic acid, and the entire tocopherol content (as an add-up of *α*-T, *β*-T, *γ*-T, and *δ*-T labeled as T_tot_) and the antioxidant capacity of the Trolox equivalent of sprouted cruciferous seeds, as established experimentally. After the model construction stage, various analyses are used to evaluate its accuracy. Sensitivity analysis is also used to determine the effect of each of the input parameters on the target values.

## 2. Description of Modeling

### 2.1. PSO

One of the techniques for stochastic optimization is PSO which was presented by Eberhart and Kennedy [25]. The application and manual for this procedure are introduced in [26–28]. An abstract of this method is summarized in six steps which will be mentioned as follows [29].

(1) $$\begin{array}{c} v_{k}^{i}\left(t+1\right)=wv_{k}^{i}\left(t\right)+c_{1}.\mathrm{rand}\left(\;\right)\left(p_{k}^{i}\left(t\right)-x_{k}^{i}\left(t\right)\right)+c_{2}.\mathrm{rand}\left(\;\right)\left(g_{k}^{i}\left(t\right)-x_{k}^{i}\left(t\right)\right), \end{array}$$

(2) $$\begin{array}{c} x_{k}^{i}\left(t+1\right)=x_{k}^{i}\left(t\right)+v_{k}^{i}\left(t+1\right)1\leq i\leq N,\;1\leq K\leq D. \end{array}$$

*Step 1*: a majority of stochastic solutions is formed as the searching space. Assume that the searching space has two parameters: dimension (*D*) and particle number (*N*). Each possible solution is dedicated to two attributes: position and velocity of the *i*^th^ particle in iteration *k*. These particles are then “flown” through the search space of possible solutions which are indicated as follows. *Step 2*: measure the fitness between each particle in the swarm. *Step 3*: for each iteration, evaluate the particle's fitness with the best fitness acquired in the previous ones. If this value is better than the best acquired previous ones, replace the amount and location of the previous one with the current value and location, respectively. *Step 4*: evaluate some fragments together and update the finest place with the best fitness (*g*_*k*_^*i*^). *Step 5*: the pace of each fragment is increased towards its (*g*_*k*_^*i*^) and (*p*_*k*_^*i*^). This speed or acceleration is valued by an accidental term. *Step 6*: start again from step 2 based on favorable factors until a new convergence is reached.

### 2.2. ELM

An ELM can be introduced as a least square-based single hidden layer (HL) feed-forward neural network (SLFN) for two problems: regression and classification. Huang et al., for the design of an ELM, apply the kernel function instead of the HL with a huge amount of nodes [30]. Hung et al. and Pal and Deswal [31] both suggested techniques; the abstract of these methods is as follows.

Hidden neurons  (*H*), ELM for the training set (*N*), and activation function *f*(*x*) can be described as follows:

(3) $$\begin{array}{c} e_{j}=\overset{H}{\underset{i=1}{\sum }}\alpha_{i}f\left(w_{i},c_{i},x_{j}\right)j=1\cdots N. \end{array}$$

*α*_*i*_ and *ω*_*i*_ are *H*-output layers and weight vectors (WVs) of the connecting input HLs (input Ws), respectively. *x*_*j*_  indicates input variables. *C*_*i*_ indicates the *H* bias for the ith *H* neuron, and *e*_*j*_ is the output of ELM for multiple data points (*j*). The process of generating input Ws is random and is based on consecutive distribution. Via a linear function, the output and result of Ws are calculated which are as follows:

(4) $$\begin{array}{c} \beta =A†Y. \end{array}$$

*A* shows the output of the HL matrix (equation (5)), A † indicates the inverse of *A* when using the Moore-Penrose method, and *Y* represents the values that ELM tries to reach. The compact and simplified form of equation (4) is  *Aα* = *Y*. *A* is the matrix of HL in the neural network (NN) and *Y* is the vectors of the output variable. The three matrixes,  *A*, *α*, and *Y*, can be represented as follows:

(5) $$\begin{array}{c} A=\left[\begin{array}{c} h\left(x_{i}\right)\\ \vdots \\ h\left(x_{N}\right) \end{array}\right]=\left[\begin{array}{ccc} f\left(w_{1},c_{1},x_{1}\right) & \cdots & f\left(w_{H},c_{H},x_{1}\right)\\ \vdots & \cdots & \vdots \\ f\left(w_{1},c_{1},x_{j}\right) & \cdots & f\left(w_{H},c_{H},x_{j}\right) \end{array}\right],\;\alpha =\left[\begin{array}{c} \alpha_{1}^{T}\\ \vdots \\ \alpha_{H}^{T} \end{array}\right],\;Y=\left[\begin{array}{c} y_{1}^{T}\\ \vdots \\ y_{N}^{T} \end{array}\right], \end{array}$$

in which *h*(*x*) represents the feature mapping of the HL. Matrix *A* strongly assigns the result of the ELM algorithm. One of the traditional solutions is using NNs in the HLs in which this type of solution uses a gradient descent algorithm as represented in [31]. In order to solve ELM, the kernel function is applied, and on the other hand, for solving the kernel matrix, feature mapping is used as can be seen in the following [30]:

(6) $$\begin{array}{c} k\left(x_{i},x_{j}\right)=h\left(x_{i}\right).h\left(x_{j}\right). \end{array}$$

In this article, we attempt to comprehend the efficiency of the kernel on the Mr prediction via KELM application and integrating ELM method with PSO for designing a fresh model for predicting Mr. In the point of view of the learning rate, predictive performance, and generalization capability, ELM has better performance in comparison with normal NNs. ELM via the Moore-Penrose generalized reverse method distinguishes the Ws of the output and input layers and produces some random values for *H* biases and input Ws [32, 33]. The general structure of a NN known as SLFN (single HL feed-forward NN) included the output and input layer neurons, *m* and *n*, respectively, and also HL neurons. For instance, suppose {*X*_*i*_, *Y*_*i*_} is a training dataset, then it can be understood that the input dataset is *X*_*i*_ = [*X*_*i*1_, *X*_*i*2_, ⋯, *X*_in_] and the output dataset is *Y*_*i*_ = [*Y*_*i*1_, *Y*_*i*2_, ⋯, *Y*_*im*_] for *i* = 1, 2, ⋯, *n*. *m* is the number of training samples.

## 3. Data Gathering

There are 172 data points in the database utilized in the present study [34], with two train (129 data points (about 75%)) and test (43 data points (about 25%)) datasets for training and testing the efficiency of proposed models, respectively. To boost the efficiency of the study models, the data points were normalized between −1 and +1.

## 4. Results and Discussion

The efficiency analysis needs to be carried out for evaluating the capability of the model. Accordingly, various statistical analyses were carried out between the actual values and the model outputs, including standard deviations (STD), mean relative errors (MRE), root mean square error (RMSE), mean squared error (MSE), and *R*-squared (*R*^2^) to evaluate the capability of the study model [35–38].

**Figure 1:**
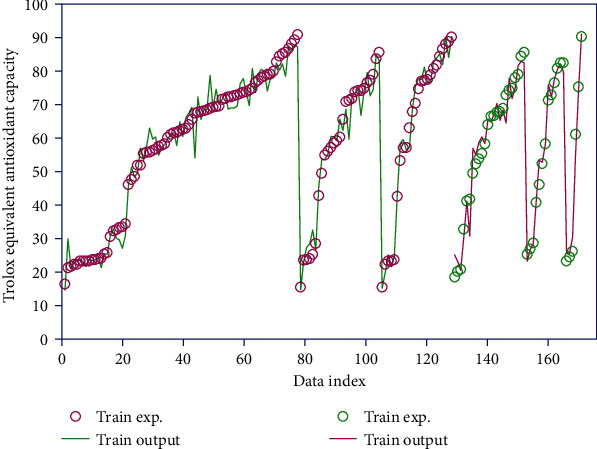
Simultaneous viewing of real and corresponding modeled data.

Figure 1 represents the actual values versus model outputs for the output data at the train and test phases. The target is accurately estimated by the study model with a decent agreement between the real information and model yields, highlighting their capability in output prediction.

**Figure 2:**
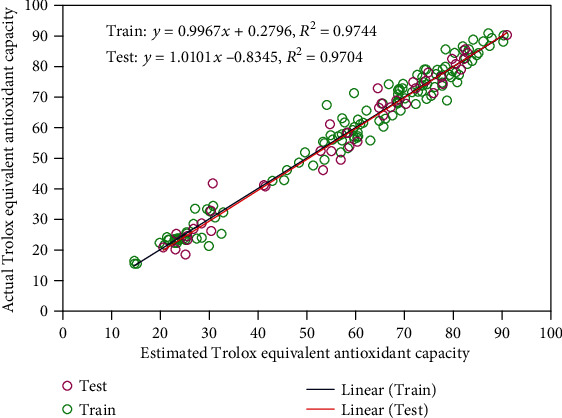
Regression analysis to evaluate the accuracy of the proposed model.

Also, the model results from regression analysis represented in Figure 2 at the train and test phases. Based on related literature, the *R*^2^ value is an eminent statistic indicating the model output-actual value relationship. The basic objective was to conduct a comparative analysis between model yields and real values. The accuracy of the model's accuracy is improved when the fitted line approaches the bisector line [39, 40]. A remarkable linear correlation is achieved between the model outputs and actual values for *R*^2^ = 1, which gets weaker when the *R*^2^ value approaches to zero [41, 42]. Accuracy is represented by a close-fitting of data points around the 45° line for the prediction models. As shown in this figure, this model shows a high ability to predict target values in different phases.

Table 1 presents the results from statistical analyses of the study model based on the RMSE, MSE, STD, MRE, and *R*^2^ parameters [43, 44].

In a study with similar input data, Buciński et al. predicted TAEC values using the artificial neural network (ANN) method [34]. Their model showed an accuracy of *R*^2^ = 0.931 in estimating the output data in the testing phase, which was weaker than the model proposed in this paper.

**Figure 3:**
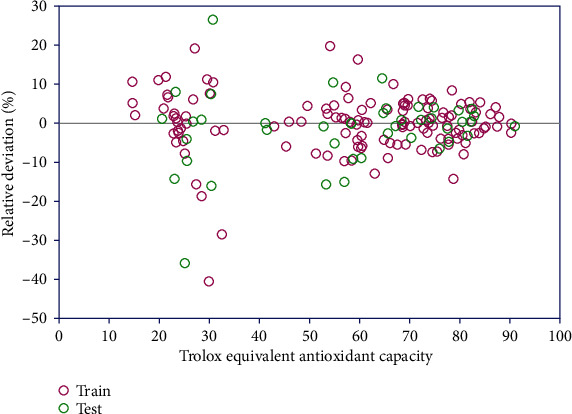
Determining relative deviation values to evaluate the accuracy of the model in predicting the target data.

Furthermore, Figure 3 represents the absolute relative deviation between the actual values and model yields of output anticipated utilizing the examination model.

**Figure 4:**
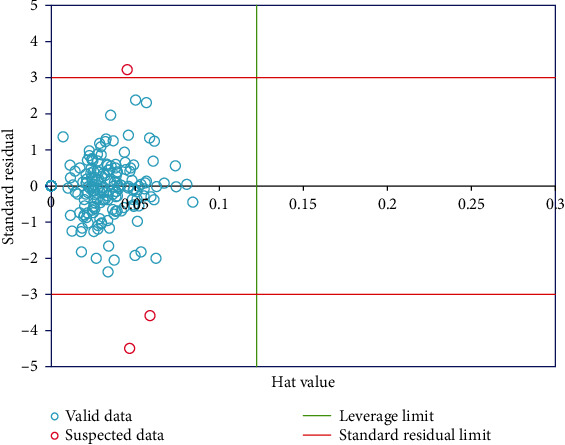
Detection of suspicious points using William's plot.

William's plot was utilized for determining the outliers of the model [45, 46].  Figure 4 represents the standardized residuals versus hat values. This figure clearly shows three limited boundaries: leverage limit, upper limit, and down suspected limit [47]. Outliers are data with higher standardized residual values > 3 or <−3, and the data with hat > hat∗ (referred to as the warning leverage value) are beyond the applicability domain of the study model [48]. As can be seen from this figure, among all the data points, only three suspicious points are seen.

**Figure 5:**
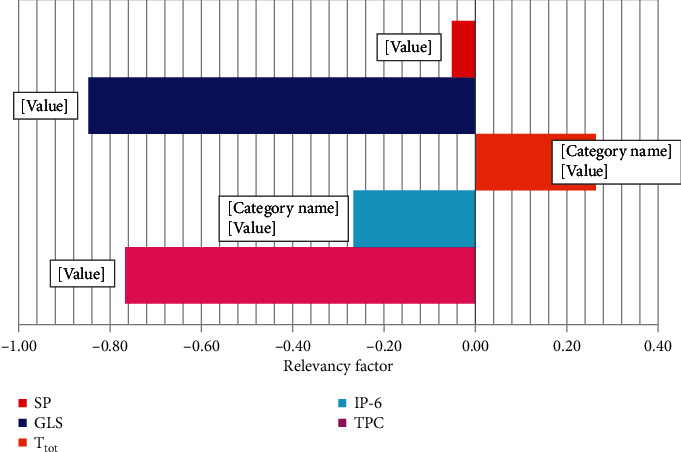
Sensitivity analysis on various input parameters of the model.

Finally, sensitivity analysis was used to determine the effect of different input parameters on the target parameter. More details about this analysis are given elsewhere [49, 50]. According to Figure 5, it was found that T_tot_ has the most direct effect on the target parameter, which corresponds to the relevancy factor (*r*) equal to +0.26, while other input parameters showed an inverse effect on the target parameter so that GLS showed the most negative effect with *r* equal to -0.85.

## 5. Conclusion

This study was aimed at seeing how well a statistical learning-based model could predict the antioxidant capacity of cruciferous sprouts. To this end, the PSO was implemented in the ELM model. When it came to setting the tuning parameters, the PSO algorithm showed good performance. Estimates were found to be quite accurate when compared to actual data points. The efficiency of the proposed techniques was verified by an excellent agreement achieved between the model outputs and the actual values in assessing the model during the train and test phases, as demonstrated by results from statistical analyses. The models' accuracy was confirmed as predicted by a comparison which was made between the proposed models' outcomes and another reported correlation. The proposed strategy to predict the antioxidant capacity of cruciferous sprouts is user-friendly so that they can be considered a useful tool for researchers, particularly in related fields, unlike the sophisticated mathematical techniques developed for this output prediction.

## Figures and Tables

**Table 1:** Determining the values of different statistical parameters for the model in different phases.

Phase	*R* ^2^	MRE (%)	MSE	RMSE	STD
Train	0.974	5.06	12.37	3.52	2.38
Test	0.970	5.90	13.65	3.70	2.60
Total	0.973	5.27	12.69	3.56	2.43

## Data Availability

The data used to support the findings of this study are provided within the article.
